# Applicable Scenarios, Desired Features, and Risks of AI Psychotherapists in Depression Treatment From the Patient’s Perspective: Exploratory Qualitative Study

**DOI:** 10.2196/85138

**Published:** 2026-05-01

**Authors:** Chunyi Xian, Aihua Yan, Yaxian Wang, Eileen Yuk Ha Tsang, Lei Huang, David (Jingjun) Xu

**Affiliations:** 1 Department of Information Systems, City University of Hong Kong, Hong Kong, China (Hong Kong); 2 Department of Marketing and International Business, Lingnan University, 8 Castle Peak Road, Tuen Mun, NT, Hong Kong, China (Hong Kong), 852 26067930; 3 Department of Social and Behavioural Sciences, City University of Hong Kong, Hong Kong, China (Hong Kong); 4 Department of Psychiatry, Tongji Hospital, Tongji University, Shanghai, China

**Keywords:** artificial intelligence, virtual therapist, mental health care, depression, user-centered

## Abstract

**Background:**

Depression is a pervasive global mental health issue, yet access to trained professionals remains severely limited. With the rapid advancement of artificial intelligence (AI), digital tools are increasingly seen as a viable way to address this shortage. However, questions remain about how digital platforms for mental health care can be effectively designed.

**Objective:**

This study aimed to investigate, from an end user’s (patient’s) perspective, the potential use scenarios, desired features, and perceived risks of AI psychotherapists in depression treatment, providing design guidelines for their development.

**Methods:**

A grounded theory approach was applied to analyze qualitative responses from 452 individuals recruited via Amazon Mechanical Turk. Data were collected through a scenario-based online survey on AI-assisted depression treatment administered between March 2023 and May 2023. Participants responded to 3 open-ended questions regarding the potential use of AI in treating depression, the characteristics expected from an AI psychotherapist, and the associated perceived risks, along with demographic, control, and contextual measures. The open-ended responses were inductively coded into themes, with intercoder reliability established (Cohen κ=0.80). In addition, variations in themes were further examined across participant profiles, including social stigma, current depression severity, trust in an AI psychotherapist, and privacy awareness.

**Results:**

Participants envisioned AI psychotherapists across 5 primary scenarios: diagnosis, treatment, consultation, self-management, and companionship. Key desired features include professionalism, warmth, precision care, empathy, remote services, active listener, personalization, flexible treatment options, patience, trustworthiness, and basic treatment alternative, while critical concerns include diagnostic inaccuracy, treatment errors, privacy breach, lack of human interaction, technical malfunctions, and lack of emotional engagement. Based on these findings, a general MoSCoW (must have, should have, could have, and won’t have) prioritization framework was proposed to serve as a conceptual starting point for future AI system design and empirical validation in mental health care. Notably, feature prioritization varied across user profiles: individuals with higher stigma placed greater emphasis on privacy protection, those with more severe depression prioritized precision care and timely access, low-trust users de-emphasized remote services, and privacy-sensitive individuals showed reduced preference for features requiring extensive data disclosure. These patterns highlight the need for context-sensitive design.

**Conclusions:**

This study provides a patient-centered framework for designing AI psychotherapists and complements the existing literature by highlighting the importance of balancing clinical effectiveness with relational considerations. The findings offer actionable guidelines for designing AI mental health care tools that are aligned with user expectations and sensitive to individual differences.

## Introduction

### Background

Depression is a major global health care challenge that affects millions of people. The growing prevalence of depression strains mental health care systems, particularly due to a shortage of qualified professionals. The imbalance between rising demand and limited resources has prompted researchers to explore alternative solutions, such as using digital technologies to support diagnosis and treatment [1-3].

Artificial intelligence (AI) is widely discussed as a potentially powerful tool for depression treatment, given its capability to improve care access and quality. Early digital tools mainly offered basic remote therapy, while modern AI can offer more sophisticated and scalable mental health care interventions. AI-empowered chatbots, virtual therapists, and predictive algorithms are already being used to deliver cognitive behavioral therapy and other forms of support [4,5]. Another key strength of AI lies in its capacity to provide consistent diagnoses for identical symptoms, in contrast to human physicians who may offer differing interpretations of the same clinical signs due to variations in their knowledge, skills, and experiences [6]. This aspect of AI systems has been argued to suggest potential in avoiding diagnostic inaccuracies and standardizing patient care across various clinical settings. Nevertheless, AI is not a replacement for therapists. In clinical settings, this technology typically takes a supportive role in decision-making, improving workflow, and managing large volumes of patient data [7]. Human oversight remains essential throughout the process.

Despite rapid technical advances and increasing adoption of AI in mental health care, patients’ expectations of AI’s role in depression treatment remain largely unknown. Most of the published work focuses on system development or clinical integration [8,9], and only a handful of studies have explored the clinical experience of care recipients. This gap could be especially problematic in mental health care contexts, where the therapeutic relationship is deeply personal and the patient is not just a passive recipient but also a central agent in the treatment process. Therefore, neglecting the patient’s voice in the design of AI-based mental health care tools may lead to a misalignment between technological function and actual user needs, and such a mismatch can result in dissatisfaction, mistrust, and ultimately the nonadoption of AI systems.

Moreover, AI in mental health care must navigate a sensitive terrain where a tone-deaf response or clunky interface may be perceived as harmful or distressing by users. For someone in a depressive episode, a poor AI interaction might feel impersonal or, even worse, harmful. This study addresses the research gaps by adopting a patient-centered approach grounded in qualitative inquiry, drawing directly from the narratives of individuals who may have symptoms of depression. Specifically, this work explores patients’ actual expectations for AI functions and qualities as well as their concerns about potential risks associated with AI. Organizing these insights through grounded theory can align with patients’ needs and enable the development of AI tools that are both technically robust and emotionally attuned.

### Related Work

Existing studies have examined AI applications across mental health care, ranging from monitoring patient states and detecting early warning signs to supporting diagnosis and treatment.

AI can assist mental health care professionals in understanding a patient’s condition by collecting and analyzing real-time data, thereby enabling more accurate diagnoses and personalized treatment plans [10,11]. Through smartphones and wearable devices, AI continuously monitors a patient’s mental state by tracking diverse indicators, such as sleep patterns, physical activity, social interactions, and subtle behavioral cues [3,12,13]. These comprehensive data provide a complete picture of an individual’s mental state [11].

AI tools also facilitate the early detection of relapse or worsening mental conditions, thereby allowing for timely intervention and improved treatment outcomes [14]. AI supports early detection by analyzing diverse sources, including patient-reported data [15], clinical notes [16], mobile use patterns [17], social media content [18], and patient messaging data [19], hence allowing algorithms to predict depression risks.

AI can also support decision-making in mental health care [20-22]. When human professionals are unavailable, AI monitors patient data over time and adjusts clinical recommendations accordingly [23]. This tool can act as an assistant to clinicians by handling administrative tasks and enhancing efficiency [24] or provide an additional safeguard by supervising clinical processes [25] and detecting nuanced psychotherapeutic information [26]. In some cases, AI can surpass human capabilities by integrating information from multiple sources to avoid diagnostic inaccuracies [27].

Some platforms also use AI to simulate therapeutic conversations and provide personalized support. For instance, Woebot and Wysa deliver cognitive behavioral therapy for conditions such as depression and anxiety. Similarly, chatbots [4] and virtual therapists [5,28] assist users in navigating various mental health care challenges.

While a substantial body of research has documented the capabilities of AI in mental health care, these studies primarily reflect the perspectives of mental health care professionals or system designers and largely ignore the voices of patients. Existing research has yet to adequately explore how patients perceive AI psychotherapists, specifically the features they value, the risks they fear, and the functions they expect. This gap warrants further exploration as patients’ acceptance, satisfaction, and comfort play a crucial role in the perceived usability and acceptability of AI systems in mental health care. This study addresses this gap by grounding the investigation in patients’ own narratives.

## Methods

### Ethical Considerations

The study was reviewed and approved by the Human Subjects Ethics Sub-Committee at City University of Hong Kong (project 11500322). Prior to participation, individuals viewed an informed-consent page outlining the study’s purpose, expected time commitment, voluntary nature of participation, types of data collected (survey responses and demographic measures), and the research team’s contact information. Only participants who provided consent were allowed to proceed to the questionnaire. No directly identifying information (eg, names, email addresses, or other personal identifiers) was collected as part of the survey. Survey responses were not linked to personally identifying information and were analyzed in deidentified form to protect participant confidentiality. Data were stored on the researcher’s local device, with access restricted to the research team. Each participant received US $5 via Amazon Mechanical Turk (MTurk) upon completing the study.

### Data Collection

Participants were recruited through MTurk, yielding a closed convenience sample of adults who voluntarily opted into the study. Data were collected between March 2023 and May 2023. Participants read a scenario in which an AI system was used to assist in depression treatment and then completed a questionnaire. Our questionnaire consisted of 3 open-ended questions, along with a set of demographic, control, and contextual items. The questions were iteratively refined to improve clarity and face validity through internal review by the research team. The questionnaire was administered across multiple pages, with related questions grouped on the same page (eg, demographic items presented together). No item randomization or adaptive branching was implemented. Participants could review their responses before final submission, and item nonresponse was permitted for nonmandatory fields. The survey link was distributed to 493 individuals. A total of 452 online surveys were completed, yielding a completion rate of 91.7%. Because recruitment occurred via MTurk (a closed platform), view and participation rates based on unique site visitors were not applicable. Cookies, IP addresses, and log-file checks were not used, but duplicate participation was prevented by restricting to one submission per MTurk ID, and only completed surveys were included in the analysis. No completion-time cutoff or statistical corrections were applied because the study focused on qualitative open-ended responses. For further details regarding the study design, please refer to the CHERRIES (Checklist for Reporting Results of Internet E-Surveys) checklist provided as Checklist 1. The participants were asked the following 3 open-ended survey questions:

- Q1: What do you think about the possibility of using AI technology in depression treatment?
- Q2: Which characteristics are you looking for from a physician? In other words, how can a physician do better to help a depressed patient?
- Q3: What could be the potential risks of using AI technology in depression treatment?

Although our Q2 used the term “physician,” all open-ended questions were explicitly framed within AI-enabled psychotherapy as defined in a vignette presented before the survey. We therefore interpreted Q2 responses as expectations for patient-valued care attributes and interaction qualities (eg, interactional behaviors, support functions, safety, and boundary setting). Our aim was to identify the desired physician attributes that are transferable to AI features. In addition to the 3 open-ended questions, we collected several control and contextual variables. We measured participants’ *computer skills* using a single question (“How would you rate your computer skills?”) [29], *familiarity with AI* using a 3-item measure (participants indicating their level of agreement with the following statements: “I am familiar with AI app”; “I am familiar with seeing a doctor using AI app”; and “I am familiar with the processes of using AI app to see a doctor”) [30], *social stigma toward depression treatment* using 5 questions (“I believe that receiving treatment for depression carries social stigma”; “I believe that it is a sign of personal weakness or inadequacy to receive treatment for depression”; “I think that people will see a person in a less favorable way if they come to know that he/she has received treatment for depression”; “I believe that it is advisable for a person to hide from people that he/she has been treated for depression”; and “I think that people tend to like less those who are receiving professional help for depression”) [31], *trust in an AI doctor* using 1 question (“To what extent do you trust an AI virtual doctor for depression treatment?”) [32], and *privacy awareness* using one single question (“In general, I care about my privacy and view the violation of my privacy seriously”) [33]. All of the above measures were assessed using 7-point Likert scales and adapted from the extant literature. We also measured participants’ *current depression severity* using the Patient Health Questionnaire-9 [34]. All continuous variables were calculated by averaging the ratings of the associated measurement items. Moreover, participants reported their demographic information, including sex, age range, and education level.

### Data Analysis

We conducted a qualitative analysis of the textual data, integrating each participant’s 3 open-ended responses to develop a holistic profile of an AI psychotherapist. More specifically, responses of Q1 were used to identify the clinical scenarios of use, responses of Q2 were used to elicit transferable care attributes, and responses of Q3 were used to capture perceived risks. The analysis followed the principles of grounded theory [35,36] and used an inductive, bottom-up approach to theme development. The first and third authors conducted the coding, while the second author randomly audited the coded data to ensure accuracy. Upon receiving the participants’ responses, coder 1 (the first author) conducted an initial open coding process, assigning preliminary labels to the raw textual data. The purpose of this step was to transform unstructured responses into manageable analytical units and to ensure that the labels comprehensively captured and accurately reflected the essence of the participants’ statements. Throughout the coding process, coder 1 set aside prior assumptions and focused on meanings emerging directly from the data [37]. This approach supported the discovery of meaningful themes suited to the exploratory nature of the study. Data saturation was reached when no additional labels emerged, and key terms and phrases related to *scenarios*, *desired features*, and *perceived risks* had been comprehensively identified. Subsequent responses largely repeated existing patterns, thereby reinforcing the established labels. Coder 1 then grouped related codes into broader thematic categories. After the themes were finalized, each theme was clearly defined to delineate its conceptual boundaries and clarify what content fell within its scope.

Subsequently, coder 1 assigned binary codes to each response for every theme (1=“theme present” and 0=“theme absent”). A codebook encompassing all identified *scenarios*, *features*, and *risks* was developed to facilitate subsequent cross-checking between coders. When coding “*features*,” coder 1 only categorized expectations as AI psychotherapist features when the response described a capability or interactional behavior that could plausibly be delivered by an AI system (or an AI component in hybrid care), rather than assuming that desirable human clinician qualities are automatically transferable to AI. Coder 2 was then provided with the raw responses and the finalized list of themes along with their definitions. Independently, coder 2 assigned binary codes to each response based on these definitions. Intercoder reliability was assessed by calculating the average matching rate across all responses (a match was defined as both coders assigning a value of 1 to the same theme). The Cohen κ coefficient yielded a value of 0.80, which falls within the range for substantial agreement (0.61‐0.80) and supports the overall consistency and validity of the coding process [38].

In addition to extracting general desired features and perceived risks, we examined how these features and risks varied across participant profiles based on (1) social stigma toward depression treatment, (2) current depression severity, (3) trust in an AI doctor, and (4) privacy awareness. Participants were first categorized into groups for each profile variable. For stigma, trust, and privacy awareness, participants were grouped into high, medium, or low bands using 1‐SD cutoffs around the mean; depression severity followed established Patient Health Questionnaire-9 criteria [34] to categorize participants into 5 levels (none, mild, moderate, moderately severe, and severe). Within each subgroup, we recalculated the frequency of each desired feature and perceived risk. These frequency distributions allowed us to identify the prioritization patterns of expected features and perceived risks across different participant profiles.

## Results

### Data Sample

The sample was relatively balanced by sex, skewed younger, and predominantly well-educated. The participants also reported moderately high levels of computer proficiency. Detailed demographic characteristics are presented in Table 1.

**Table 1.** Participant demographics and computer proficiency (N=452).

Variables			Values	
Categorical variables, n (%)				
Sex				
Male			226 (50)	
Female			225 (49.8)	
Other			1 (0.2)	
Age group (y)				
<18			11 (2.4)	
18‐24			134 (29.6)	
25‐34			145 (32.1)	
35‐44			83 (18.4)	
45‐54			62 (13.7)	
55‐64			17 (3.8)	
Education level				
Some school, no degree			3 (0.7)	
High school diploma			39 (8.6)	
Some college, no degree			77 (17)	
Bachelor’s degree			257 (56.9)	
Master’s degree			66 (14.6)	
Professional degree			7 (1.5)	
Doctorate degree			3 (0.7)	
Continuous variables, mean (SD; range)				
Computer skill			5.45 (1.07; 2-7)	
Familiarity with AI^a^			4.39 (1.81; 1-7)	
Social stigma			4.29 (1.62; 1-6.8)	
Depression level			9.85 (7.43; 0-27)	
Trust in AI doctor			4.30 (1.64; 1-7)	
Privacy awareness			5.69 (1.18; 1-7)	

a AI: artificial intelligence.

### Key Scenarios for the Application of AI Psychotherapists

Five envisioned scenarios of AI psychotherapist applications emerged from the collected responses, including diagnosis, treatment, consultation, self-management, and companionship (Table 2). The most frequently mentioned scenario was diagnosis, followed by treatment and consultation, indicating a clear emphasis on the clinical and decision-support functions of AI psychotherapists. Self-management reflects expectations for AI tools to assist users in independently managing their mental health over time. While less prominent, companionship suggests that some participants envision AI playing an emotional role. Taken together, these scenarios suggest that the participants imagined AI functioning across a broad spectrum of care, ranging from diagnostic assessment to emotional companionship.

**Table 2.** Artificial intelligence (AI) psychotherapist scenarios mentioned by the participants.

Scenario	Frequency	Description	Sample quotes
Diagnosis	273	Use AI to analyze symptoms and generate diagnostic conclusions	“AI has the potential to help clinicians better understand and diagnose depression.”
Treatment	229	Use AI to deliver behavioral and psychological interventions	“The potential use of AI technology in depression treatment is an exciting prospect.”
Consultation	190	Use AI to conduct consultation sessions and provide mental health care advice.	“People (who) are hesitant about getting treatment...can first talk to an impersonal AI.”
Self-management	146	Use AI to track mood and stress levels, and perform regular mental state check-ups.	“AI technology could also potentially help to identify early warning signs of depression and provide preventative measures.”
Companionship	72	Use AI to offer a supportive presence and understanding.	“AI technology could also help to provide emotional support and comfort.”

### Expected Features of AI Depression Psychotherapists

Expected features were identified through interpretive coding. Some of these features were explicitly stated (eg, “I want [feature]” or “It should [do something]”), while others were inferred from negative statements (eg, “I would avoid an AI doctor that [does something]”). In such instances, undesirable attributes were conceptually reframed as their positive counterparts to inform design features aimed at mitigating user resistance. These features reflect participant expectations rather than empirically validated requirements. A broad range of expected features was identified using a criterion of at least 5% of the 452 participants (ie, mentioned ≥23 times). This threshold was used to highlight the most salient features that may warrant prioritization in AI psychotherapist implementation. These features are summarized in Table 3. Sample quotes for all the expected features are provided in Multimedia Appendix 1.

**Figure 1.**
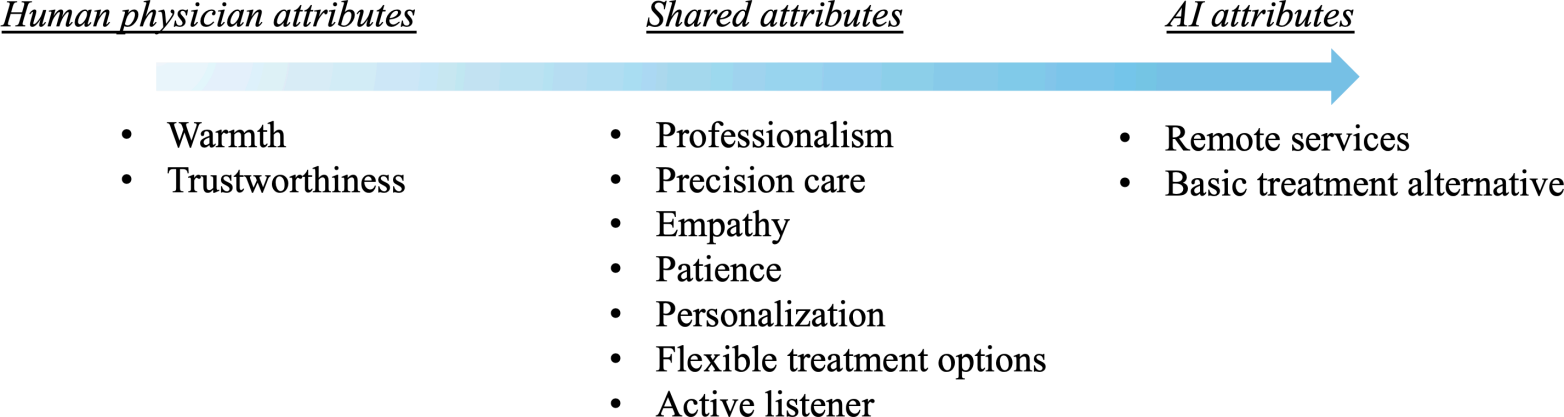
Continuum of desired attributes from human physician to artificial intelligence (AI) psychotherapist.

Participants’ expectations can be mapped onto a continuum of desired attributes, ranging from those traditionally associated with human physicians, through a middle ground of qualities that both human physicians and AI psychotherapists can potentially possess, to attributes that primarily apply to AI psychotherapists (Figure 1). Importantly, attributes located in the shared middle region do not imply equivalence between AI and human physicians; rather, they indicate domains in which AI psychotherapists may approximate valued aspects of clinical interaction through deliberate design, structured workflows, and adaptive responses. Despite this continuum, we retain and discuss the full set of desired attributes because participants framed them as a holistic profile of “good care,” which we henceforth treat collectively as expected features of an AI psychotherapist. In this sense, even attributes that sit at the human physician end of the continuum remain informative for AI psychotherapist design and implementation: they can be applied in hybrid AI-human care models, where the human component handles relational aspects, while AI contributes complementary strengths.

**Table 3.** Key features of artificial intelligence (AI) depression psychotherapists anticipated by the participants.

Expected features	Frequency	Description	Convergent with prior work
Professionalism	104	Built with guidance from mental health care professionals.	[39]
Warmth	103	Responds with friendly and emotionally supportive language to create a welcoming and comfortable interaction.	[5,28]
Precision care	101	Delivers highly accurate, data-driven treatment.	[14]
Empathy	93	Responds with deep emotional understanding.	[5,28]
Remote services	60	Access care from anywhere, improving convenience and accessibility.	[5]
Active listener	44	Attentively engage with the patient, respond appropriately, and ask effective follow-up questions to deepen the conversation.	[28]
Personalization	42	Avoid applying a one-size-fits-all approach; tailor responses, plans, and treatments to individual patients.	[5]
Flexible treatment options	28	Provide multiple treatment pathways and allow patients to choose according to their preferences rather than imposing a single fixed approach.	New feature
Patience	28	Avoid rushing; allow sufficient time for thorough symptom reporting and for patients to ask detailed questions.	[28]
Trustworthiness	27	Ensure the AI provides confidence in treatment efficacy and safeguards privacy.	[5,28,40]
Basic treatment alternative	23	Use AI to perform basic check-ups, management, and registration but not to replace complex clinical processes.	[5]

### Perceived Risks of AI Depression Psychotherapists

Perceived risks were also identified through an interpretive coding process. In addition to explicitly naming risks, the participants described specific situations they feared. In such cases, the concrete examples were abstracted into higher-level categories based on their underlying nature (whether rooted in the diagnostic process, the therapeutic interaction, and so on). As with the analysis of expected features, the present focus is limited to risks mentioned by at least 5% of the participants to highlight the most salient concerns. These risks are summarized in Table 4, and sample quotes corresponding to each perceived risk are provided in Multimedia Appendix 1.

**Table 4.** Key risks of artificial intelligence depression psychotherapists as perceived by the participants.

Perceived risks	Frequency	Description	Convergent with prior work
Diagnostic inaccuracy	165	Inaccurate conclusions about the type or name of an illness due to limited contextual information or the absence of nonverbal cues.	[11,39,41]
Treatment errors	99	Incorrect interventions after an illness has been confirmed, such as prescribing an unsuitable therapy approach or unsafe medication choices.	[3,40]
Privacy breach	91	Unauthorized access, data leaks, or misuse of personal health care information.	[3,6,11,13]
Lack of human interaction	47	Absence of physical presence, touch, or direct interpersonal connection with human beings during the interaction.	[5,28]
Technical malfunctions	42	System fails to operate properly, such as server crashes or shutdowns that prevent service delivery.	New risk
Lack of emotional engagement	32	Absence of emotionally supportive words, language, or tone.	[5,28]

### General MoSCoW Prioritization Framework

**Figure 2.**
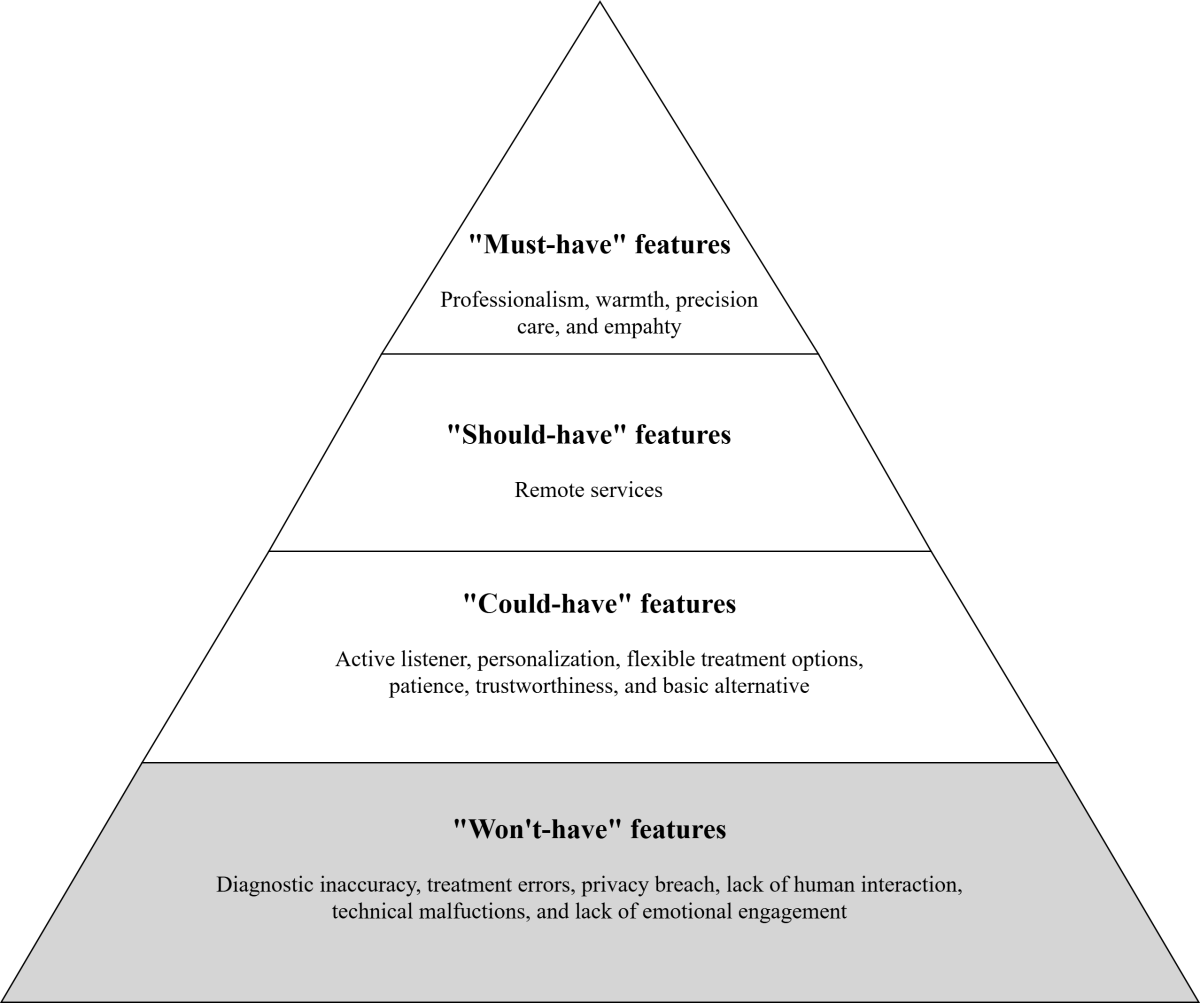
General MoSCoW (must have, should have, could have, won’t have) prioritization framework.

To translate participants’ expected features and perceived risks into a conceptual prioritization structure, we applied the MoSCoW framework [42] and used mention frequency as an indicator of salience. Features mentioned by at least 20% of participants were classified as “Must-have,” those mentioned by 10% to 20% as “Should-have,” and those mentioned by 5% to 10% as “Could-have.” In addition, the “Won’t-have” category captures risks and undesirable aspects that the system should explicitly avoid to prevent exacerbating users’ primary concerns. The resulting general MoSCoW prioritization is summarized in Figure 2.

### Differences in MoSCoW Prioritization Across User Profiles

#### Overview

**Figure 3.**
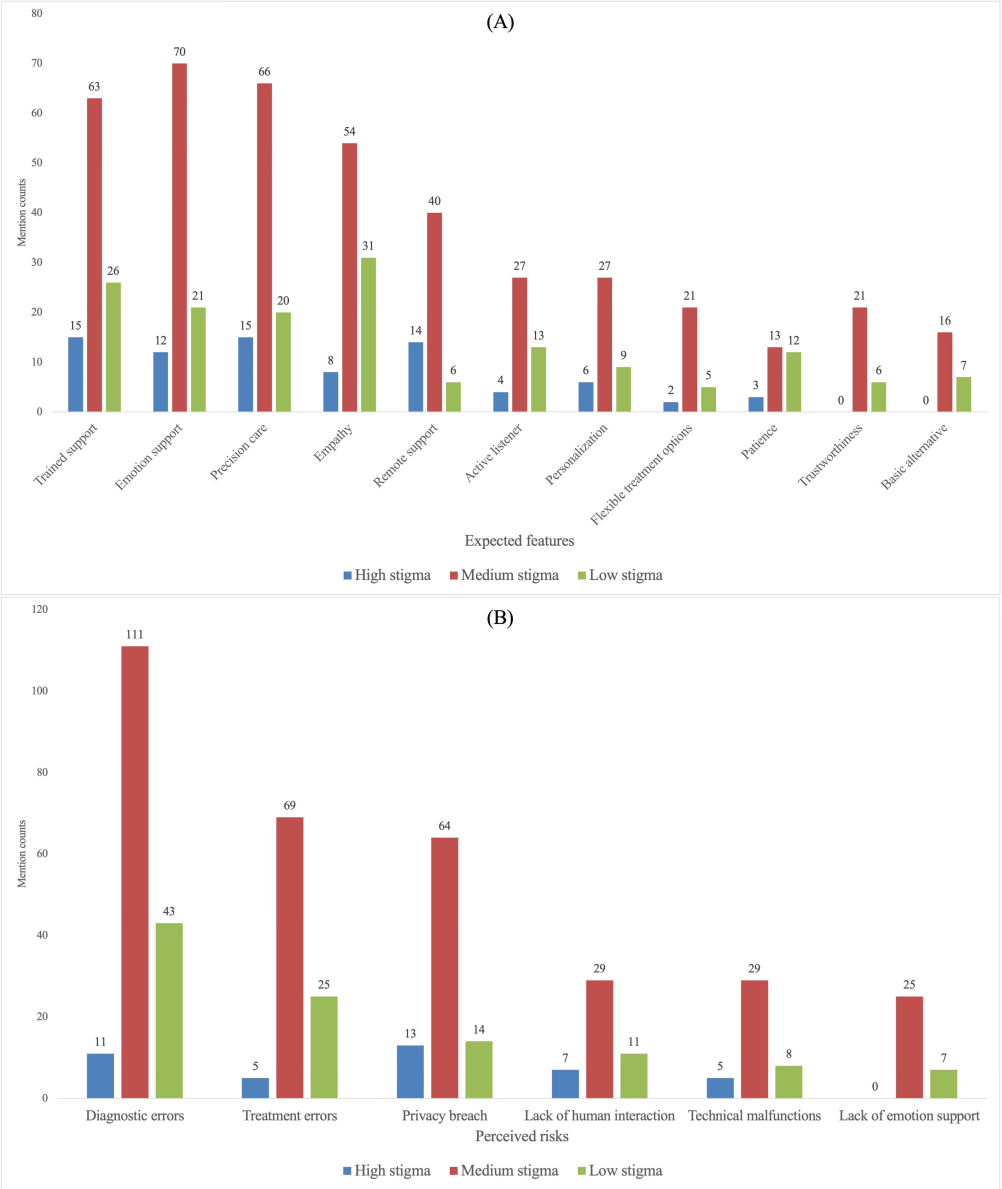
Comparing stigma levels on (A) expected features and (B) perceived risks mention counts.

**Figure 4.**
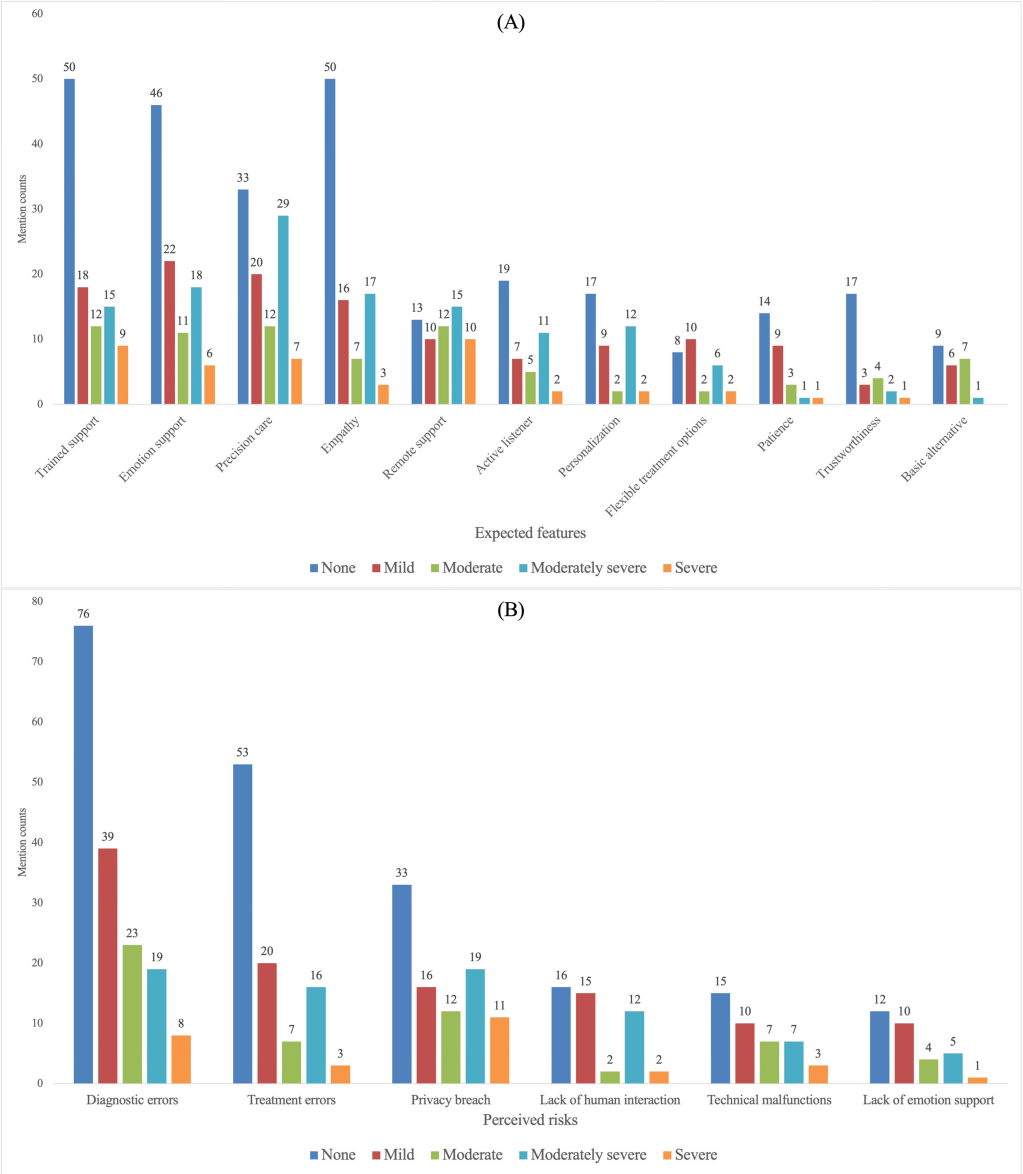
Comparison of depression severity groups on (A) expected feature and (B) perceived risk mention counts.

**Figure 5.**
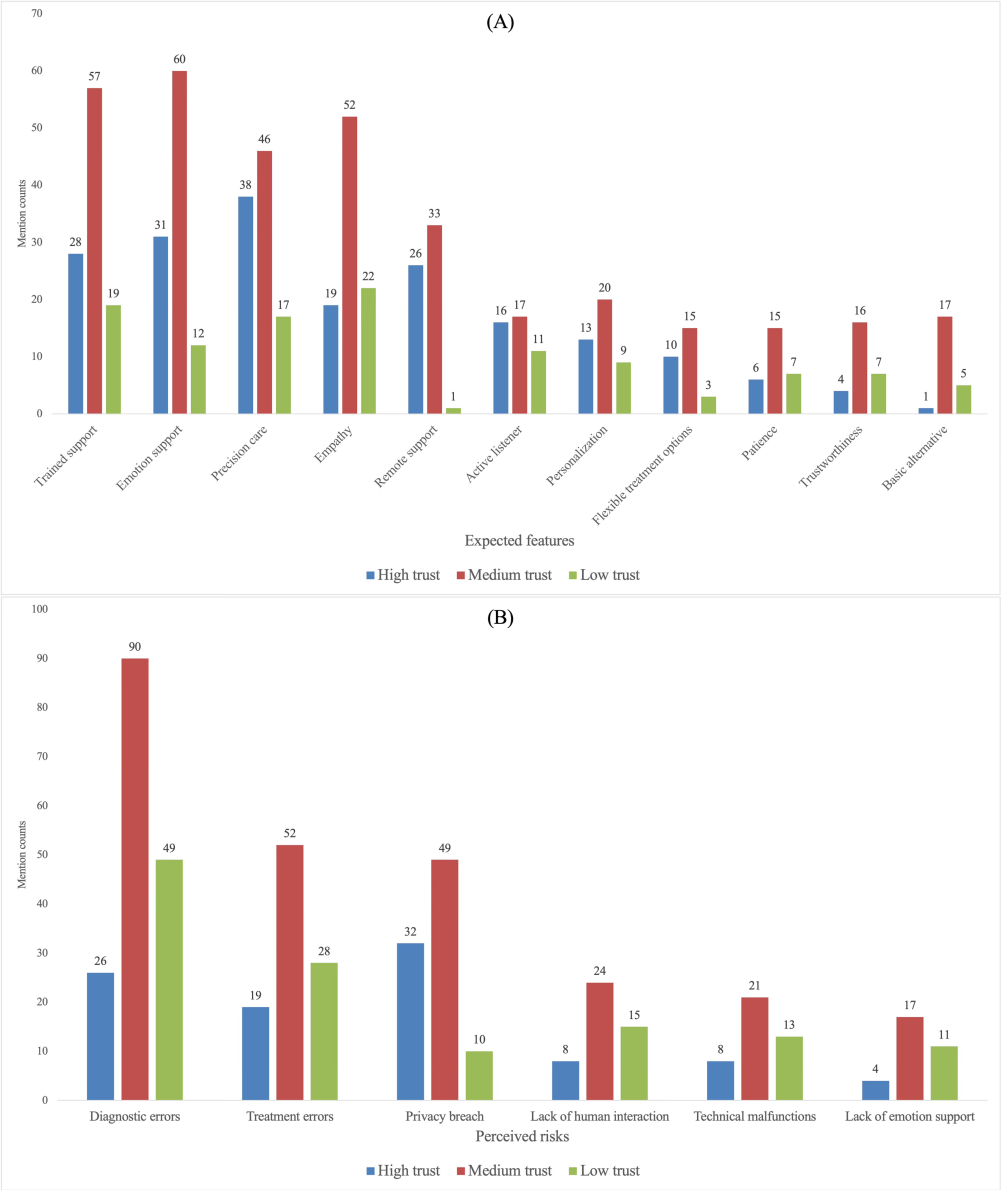
Comparison of trust groups on (A) expected features and (B) perceived risks mention counts.

**Figure 6.**
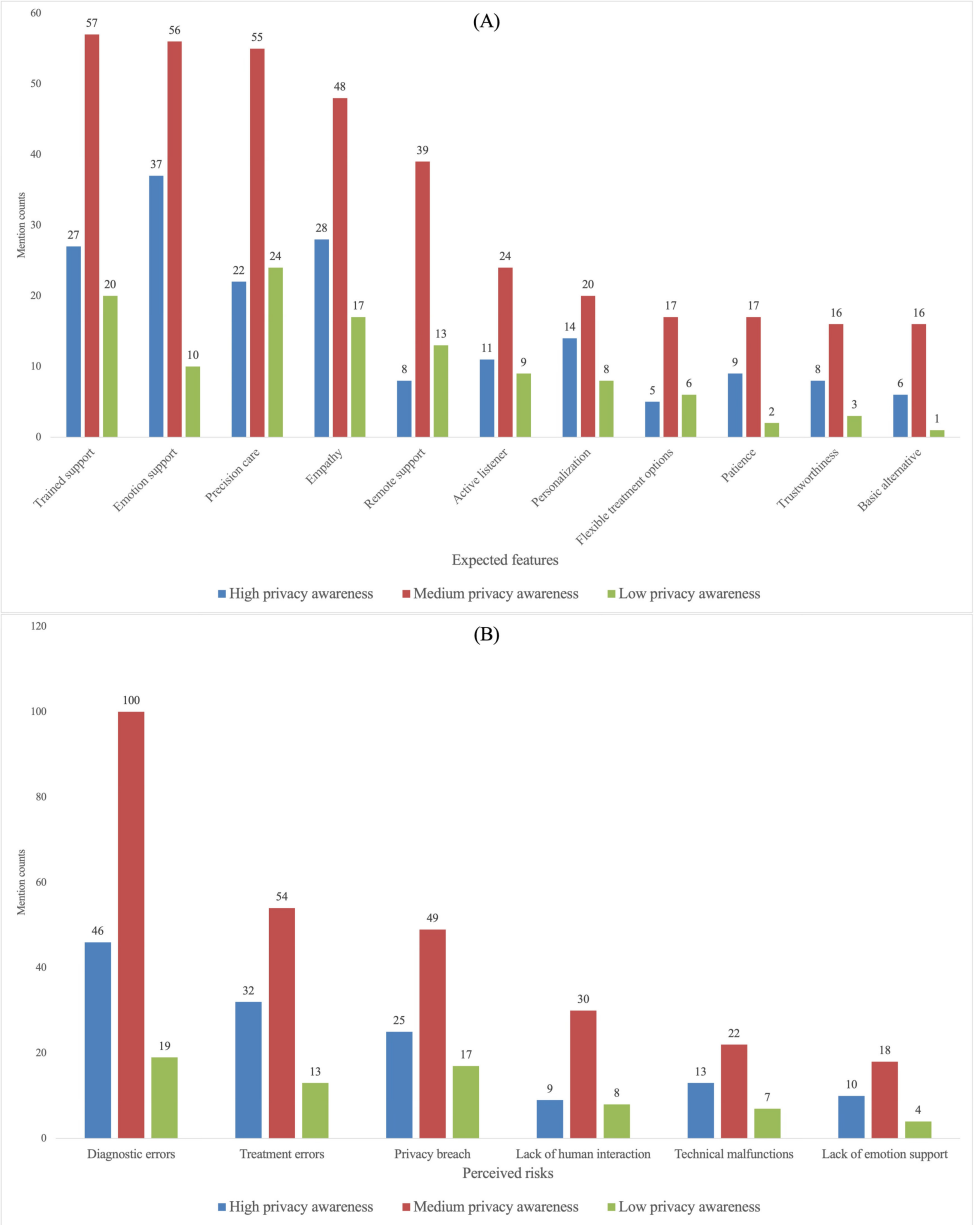
Comparison of privacy awareness groups on (A) expected features and (B) perceived risks mention counts.

While Figure 2 presents the overall prioritization, we next examined whether expected-feature and perceived-risk mentions varied across participant profiles related to (1) social stigma toward depression treatment, (2) current depression severity, (3) trust in an AI doctor, and (4) privacy awareness. The subgroup comparisons are visualized in Figures 3-6. When subgroup patterns closely mirrored the overall prioritization, no special designation was applied. However, when a subgroup exhibited substantial deviations—such as attributes or risks being unusually frequently prioritized or unusually de-emphasized relative to the general pattern—these differences were descriptively reported in this Results section and subsequently interpreted in the Discussion section.

#### Social Stigma Toward Depression Treatment

While the medium- and low-stigma groups largely followed the overall prioritization pattern, the high-stigma group showed a distinct emphasis on risks (Figure 3). In particular, compared with diagnostic inaccuracy and treatment errors, high-stigma participants placed relatively greater emphasis on preventing privacy breach, indicating heightened salience of confidentiality-related concerns in this subgroup.

#### Current Depression Level

Participants without depression assigned comparatively lower priority to precision care (Figure 4). Participants with moderately severe depression emphasized precision care more strongly. The mild and moderate groups mentioned basic treatment alternative more frequently than the general pattern, whereas the severe group emphasized remote services to a greater *e*xtent.

#### Trust in AI Psychotherapists

Expected-feature and perceived-risk mentions also varied across trust levels (Figure 5). Compared with the general pattern, the high-trust group placed relatively less emphasis on mitigating interactional deficits (eg, lack of human interaction and lack of emotional engagement). By contrast, the low-trust group assigned comparatively lower priority to remote services.

#### Privacy Awareness

Participants with higher privacy awareness placed comparatively lower priority on remote services and flexible treatment options, suggesting a reduced emphasis on capabilities that typically require increased disclosure and user interaction to function effectively (Figure 6).

## Discussion

### Principal Findings

#### Overview

This study adopted a patient-centered perspective to investigate the applicable scenarios, expected features, and perceived risks of AI psychotherapists in depression care. The findings reveal that end users may imagine an AI psychotherapist‘s application in the 5 main scenarios, including diagnosis, treatment, consultation, self-management, and companionship. Furthermore, guided by the MoSCoW prioritization framework, this study constructs a structured hierarchy from the frequency of respondents’ mentions regarding AI psychotherapist features and risks. This hierarchy serves as the basis for a deeper discussion of the divergent priorities and concerns that characterize different user segments.

#### General MoSCoW Prioritization Framework

The results translate patient-stated expectations and concerns into a MoSCoW hierarchy (Figure 2), which can inform early-stage design reasoning and future empirical testing of AI psychotherapists.

“Must-have” features represent features that participants consistently viewed as nonnegotiable in a hypothetical AI psychotherapist. Professionalism, warmth, precision care, and empathy emerged as the core attributes of AI depression psychotherapists. These qualities are perceived by participants as signals of accuracy and professional grounding [14,39]. Furthermore, the emphasis on warmth and empathy reflects a preference for AI-deliverable supportive interaction cues (eg, validating tone, attentive responses, and respectful language) that help patients feel genuinely cared for and understood [43-45]. This finding is consistent with prior research, which also highlights professional competency and emotional connection as critical factors for the acceptance and effectiveness of digital mental health interventions [45-47]. “Should-have” features are important though less critical than core functionalities. The remote services feature was included to enhance patient accessibility. “Could-have” features represent desirable enhancements that provide additional value. Active listener, personalization, flexible treatment options, patience, trustworthiness, and basic treatment alternative fall into this category*,* contributing to a more satisfactory user experience.

“Won’t-have” features are originally defined as not included in the current release but are potentially considered for future versions in software development. In this study, however, “Won’t-have” refers to aspects that an AI depression psychotherapist should explicitly avoid to prevent exacerbating users’ primary concerns. Specifically, it captures participant-identified risks and unacceptable outcomes that the system should avoid or actively mitigate, rather than merely deprioritized features. We adopt this adapted use to clearly distinguish positively desired capabilities (“Must-have,” “Should-have,” or “Could-have”) from negative outcomes that participants consistently deemed unacceptable in a hypothetical AI psychotherapist. Accordingly, “Won’t-have” is used here as a risk-avoidance category rather than a conventional feature-prioritization label. These risks include diagnostic inaccuracy, treatment errors, privacy breach, lack of human interaction, technical malfunctions, and lack of emotional engagement*.* Prior studies have similarly highlighted these risks as critical barriers to the adoption of AI-enabled mental health tools [3,41]. Notably, unlike technical errors or privacy risks, the lack of human interaction and emotional engagement is intrinsic to AI’s nonhuman nature and therefore cannot be fully “engineered away.” This implies that AI psychotherapists may be more appropriately positioned as supportive or augmentative tools within hybrid AI-human care models, in which clinicians provide emotional attunement, empathy, and relational judgment that AI cannot replicate, while AI contributes scalability, consistency, and accessibility.

#### Extended MoSCoW Framework: Factors Influencing Perceptions of AI Psychotherapists

Although the overall MoSCoW hierarchy is broadly shared, the results (Figures3^6^) show that prioritization can shift depending on user attributes that shape vulnerability, clinical need, and perceived acceptability. These differences suggest that a “one-size-fits-all” system design may be insufficient in depression-care contexts, and that targeted design emphases may be necessary for specific populations. They also point to promising directions for future system differentiation that warrant empirical testing.

##### Social Stigma Toward Depression Treatment

Stigma appears to meaningfully reweight what users view as nonnegotiable safeguards. For high-stigma individuals, confidentiality-related threats become especially salient (Figure 3) because privacy loss is not merely an informational risk—it can trigger social judgment, discrimination, and heightened vulnerability. This pattern implies that for stigma-sensitive users, foregrounding privacy as a core trust anchor (eg, clear confidentiality assurances, discreet interaction flows, and transparent data-handling boundaries) may be critically important. This conclusion is in line with previous studies, which identified that perceived stigma and fear of social judgment increase privacy protection among mental health service users [48]. In practice, emphasizing privacy-preserving defaults and making protections legible to users may be as critical as improving model performance when deploying AI tools in stigmatized care domains.

##### Current Depression Level

Differences by depression severity suggest that priorities align with clinical urgency and functional needs (Figure 4). As symptom burden increases, users may place greater weight on capabilities that plausibly affect outcomes (eg, precision care and timely access), whereas lower-severity users may evaluate AI tools more as threshold or supplemental support (eg, basic treatment alternatives and early-stage assistance). This implies that AI depression psychotherapists may require severity-sensitive positioning: systems targeted at higher-need users should emphasize professional competence, safety checks, and reliable escalation pathways, while systems intended for early-stage or milder cases may benefit from emphasizing supportive engagement and accessible, low-barrier assistance. Existing studies have explored different degrees of depression [49] and found that the same treatment approach is not suitable for varying levels of depression [50]. However, there is still a lack of in-depth research into what types of treatment are expected by patients with varying severities of depression. The findings of this study will help refine future treatment plans tailored to different depressive populations.

##### Trust in AI Psychotherapists

Trust functions as a lens through which users evaluate both benefits and deficits. When trust is high, users may tolerate limitations in humanlike interaction because they believe the system is competent and helpful [51]; when trust is low, users may discount even clearly beneficial capabilities (such as remote services) because basic reliability is in doubt (Figure 5). This implies that trust-building mechanisms are prerequisites for sustained engagement—particularly in remote or self-guided deployments. Design strategies such as transparency cues, clinically grounded explanations, conservative error-handling, and visible oversight or escalation options may help reduce skepticism and make remote delivery acceptable to lower-trust users.

##### Privacy Awareness

Heightened privacy awareness appears to dampen enthusiasm for features that typically require deeper disclosure or richer interaction to function effectively (Figure 6). As previous research has demonstrated, the majority of patients choose to trust human therapists over AI-driven systems when concerns regarding the security of personal data arise [47]. This underscores a central design tension: advanced personalization and flexible intervention often depend on sensitive data, but privacy-sensitive users may reduce disclosure precisely when systems need information to help. A viable path forward is to make privacy-preserving personalization more explicit and controllable (eg, granular consent, minimal-data modes, and clear opt-in pathways), so users can benefit from advanced functions without feeling that disclosure is coerced or opaque.

### Implications

This study leverages the mention frequency of features and risks to build a MoSCoW prioritization framework specifically for AI psychotherapists. To the best of our knowledge, it is among the first to systematically capture and synthesize patient expectations and perceived risks related to AI psychotherapists and to propose such an implementation hierarchy. Importantly, the findings reflect participants’ expectations and concerns elicited in a hypothetical context, rather than evaluations based on direct interaction with AI psychotherapists. Accordingly, the proposed MoSCoW framework should be interpreted as a foundational, user-informed prioritization schema, intended to inform future system prototyping and empirical validation, rather than as a set of validated or prescriptive design requirements.

Given the applied relevance of participants’ perspectives, the findings of this study offer several design-relevant insights for the design of AI-based tools for depression care. A key strength of this study lies in the breadth of clinical scenarios represented. As shown in Table 2, the participants envisioned AI capabilities spanning the continuum of mental health care, including diagnosis, treatment, consultation, self-management, and companionship. This versatility points to how future systems could be adapted to diverse use cases, including home-based self-assessment tools, previsit screening aids, postvisit follow-up interfaces, or in-session assistants for documentation and care planning.

Furthermore, the MoSCoW prioritization framework can serve as a conceptual reference point for designers and researchers when formulating and testing AI psychotherapist prototypes. In addition to the general prioritization applicable to broad user groups, project managers targeting specific populations with well-defined attributes can draw on the extended MoSCoW framework to tailor system features. These actionable insights offer a valuable and practical foundation for system developers and clinical stakeholders seeking to align design decisions with user-specific needs.

Ultimately, aligning AI functionalities and design with patient expectations is hypothesized to foster trust and engagement, which should be evaluated in future empirical studies. Grounding features and safeguards in user values makes the system more intuitive, credible, and meaningful, which may inform future investigations of adoption and retention.

### Limitations and Future Research

This study has several limitations. First, the study used cross-sectional online survey data. While it captured user narratives about opportunities, desired features, and perceived risks in AI-based depression treatment, it provided limited insights into the underlying reasons. The design also precluded a follow-up assessment of how satisfaction or user experience might change if an AI psychotherapist met expectations and mitigated concerns. Thus, this study provides a useful foundation but leaves user outcomes unexamined. Future research should extend this work by using qualitative methods to explore the motivations and contextual factors in depth and by using longitudinal or mixed methods approaches to evaluate the impact of these prioritized features and risk mitigations on satisfaction, trust, engagement, and behavioral outcomes. Moreover, although AI capabilities have improved since data collection, AI applications in depression treatment remain limited in real-world deployment and constrained in clinical scope. As such, the expectations and concerns captured in this study primarily reflect enduring values related to trust, safety, emotional support, and human oversight, which are less sensitive to short-term advances in model performance. These perspectives, therefore, remain relevant for informing responsible design and hybrid AI-human care models.

Furthermore, the sample was recruited via MTurk. Although MTurk is a prevalent platform for data collection in medical and psychological research, its participant demographics tend to be younger, more educated, and exhibit greater technological proficiency compared to the general population. This may limit the generalizability of the findings, particularly to older adults or individuals from diverse cultural and socioeconomic backgrounds. Future research should aim to recruit more diverse samples, including individuals from underrepresented or high-stigma groups, to enhance the external validity of the findings.

Third, the analysis used a binary coding scheme (theme present or absent) to systematically categorize open-ended responses. While this approach is easy to quantify, it may not capture the full range of themes within participants’ narratives. Moreover, we included only themes mentioned by at least 5% of participants in the primary analysis to establish a clear priority framework. This ensured that we focused on the most core user perspectives but may have overlooked some rare, yet potentially significant, insights. Future research should use in-depth qualitative methods, such as semistructured interviews or focus groups, to explore a broader range of themes.

### Conclusions

This study provides a patient-centered perspective on the use of AI in mental health care, identifying diverse scenarios envisioned by participants for the application of AI psychotherapists and prioritizing expected features and concerns based on user input. The findings highlight which qualities participants believe an effective AI depression psychotherapist should embody and emphasize the need to extend beyond clinical accuracy to address interpersonal support, emotional engagement, accessibility, and privacy protection. Designing systems in alignment with these user-informed priorities is critical to creating AI psychotherapists that are functionally effective, trusted, engaging, and responsive to the needs of users. Future work is needed to evaluate whether systems designed around these priorities lead to improved satisfaction, adherence, or clinical outcomes. As AI technologies continue to advance in mental health care contexts, embedding patient voices and values into their development will be critical to supporting their ethical integrity and guiding future real-world impact.

## Supplementary material

10.2196/85138 Multimedia Appendix 1 Sample quotes on artificial intelligence (AI) depression psychotherapist features and risks.

10.2196/85138 Checklist 1 CHERRIES checklist.

## Abbreviations

AI: artificial intelligence
CHERRIES: Checklist for Reporting Results of Internet E-Surveys
MoSCoW: must have, should have, could have, and won’t have
MTurk: Amazon Mechanical Turk
