# Supplementary material for: Applicable Scenarios, Desired Features, and Risks of AI Psychotherapists in Depression Treatment From the Patient’s Perspective: Exploratory Qualitative Study
Source: JMIR Form Res. 2026 May 1;10:e85138. doi: 10.2196/85138 (PMC13134827; doi:10.2196/85138)
Supplement: Multimedia Appendix 1 [file formative-v10-e85138-s001.docx]

**Supplementary Materials**

Appendix A. Sample quotes on AI depression psychotherapist features

| Expected features | Sample quote |
| --- | --- |
| Professionalism | *I want it (AI) to be competent and effective … It (AI) needs to be knowledgeable.* |
| Warmth | *I think there needs to be a warm … relationship, which AI cannot provide…* |
| Precision Care | *AI can help … automate repetitive tasks … AI can also help detect patterns in behavior and identify signs of clinical depression more quickly …* |
| Empathy | *Show … compassion for what the patient is going through.* |
| Remote Services | *AI technology could also be used to provide virtual support and counselling to patients, which could be especially beneficial for those who are unable to access in-person therapy.* |
| Active Listener | *… take the time to listen to the patient, ask questions to gain a better understanding of the patient’s condition and needs, provide clear and informed advice, and keep the patient updated on the progress and outcomes of treatments.* |
| Personalization | *AI technology has the potential to make personalized treatment plans tailored to an individual’s specific needs, which could be incredibly beneficial for those users with depression.* |
| Flexible Treatment Options | *… willing to consider all possible treatment options and be willing to take into account the patient’s unique situation and preferences.* |
| Patience | *… can listen to all concerns and not brush them under the rug.* |
| Trustworthiness | *… trustworthy, honest* |
| Basic treatment Alternative | *For basic treatments, it (AI) sounds great.* |

Appendix B. Sample quotes on AI depression psychotherapist risks

| Perceived risks | Sample quotes |
| --- | --- |
| Diagnostic Inaccuracy | *It (AI) could misread the symptoms and misdiagnose patients.* |
| Treatment Errors | *… How would the AI virtual doctor understand and utilize non-verbal communication?* |
| Privacy Breach | *The most obvious and direct weakness of AI in healthcare is that it can bring about a security breach …* |
| Lack of Human Interaction | *… Relying solely on AI-based interventions may result in a reduced sense of human connection …* |
| Technical Malfunctions | *AI system errors put patients at risk of injuries.* |
| Lack of Emotion Engagement | *The primary potential risk when using AI technology in depression treatment is … a lack of … emotional engagement.* |
