# Supplementary material for: Applicable Scenarios, Desired Features, and Risks of AI Psychotherapists in Depression Treatment From the Patient’s Perspective: Exploratory Qualitative Study
Source: JMIR Form Res. 2026 May 1;10:e85138. doi: 10.2196/85138 (PMC13134827; doi:10.2196/85138)
Supplement: Checklist 1 [file formative-v10-e85138-s002.pdf]

The CHERRIES checklist used here is adapted from Eysenbach G, *Improving the Quality of Web Surveys: The Checklist for Reporting Results of Internet E-Surveys (CHERRIES)*, J Med Internet Res 2004;6(3):e34. doi: 10.2196/jmir.6.3.e34

CHERRIES checklist

**1) Design**	
**Describe the survey design:** Describe target population, sample frame. Is the sample a convenience sample?	✓
**IRB approval:** State whether the study was approved by an institutional review board/ethics committee, and provide approval details if applicable.	✓
**Informed consent:** Describe the informed-consent process (what participants saw/confirmed before starting).	✓
**Data protection:** Describe how data were protected/stored and privacy/security measures.	✓
**2) Development and pre-testing**	
**Development and testing:** Describe how the questionnaire was developed and whether it was pre-tested/piloted/usability-tested.	✓
**3) Recruitment process and description of the sample having access to the questionnaire**	
**Open vs closed survey:** Specify whether the survey was open (anyone can access) or closed (restricted access).	✓
**Contact mode:** Describe how potential respondents were contacted (eg, web, email, platform invitation).	✓
**Advertising the survey:** Describe how/where the survey was advertised or posted, and what information was shown in the invitation/advertisement.	✓
**4) Survey administration**	
**Web/E-mail:** If applicable, describe whether recruitment used the web and/or email, and how links were distributed.	✓
**Context:** Describe the context in which the survey was administered (eg, website, platform, clinic, community).	✓
**Mandatory/voluntary:** State whether participation was mandatory or voluntary.	✓
**Incentives:** Describe any incentives/compensation offered.	✓
**Time/Date:** State the time period during which the survey was open / data were collected.	✓
**Randomization of items or questionnaires:** State whether item order / questionnaire order was randomized, and how.	✓
**Adaptive questioning:** State whether adaptive questioning/skip logic was used and how it worked (or state “not used”).	✓
**Number of items:** State the number of survey items (and/or sections, if relevant).	✓
**Number of screens (pages):** State the number of pages/screens (or how items were paginated).	✓
**Completeness check:** Describe any completeness checks (eg, mandatory items, prompts for missing responses, validation).	✓
**Review step:** State whether respondents could review/change answers before final submission.	✓

**5) Response rates**	
**Unique site visitor:** Define how unique site visitors were measured (if applicable).	Not applicable; as explained in the Methodology section, the survey was open to anyone visiting the website, so the total denominator (i.e., all unique site visitors who could have participated) cannot be determined.
**View rate:** Report view rate = (unique survey visitors) / (unique site visitors), and define numerator/denominator.	
**Participation rate:** Report participation rate = (unique visitors who agreed to participate) / (unique first survey page visitors), and define numerator/denominator.	
**Completion rate:** Report completion rate = (users who finished the survey) / (users who agreed to participate), and define numerator/denominator.	✓
**6) Preventing multiple entries from the same individual**	
**Cookies used:** State whether cookies were used to prevent multiple entries and how (or “not used”).	✓
**IP check:** State whether IP checking was used and how (or “not used”).	✓
**Log file analysis:** State whether log-file analysis was used to identify duplicates and how (or “not used”).	✓
**Registration:** State whether registration/login/unique IDs were used to prevent multiple entries (or “not used”).	✓
**7) Analysis**	
**Handling of incomplete questionnaires:** Explain how incomplete questionnaires were handled (excluded, partial analysis, imputation, etc.).	✓
**Questionnaires submitted with an atypical timestamp:** Explain whether responses with atypical completion times were identified and how they were handled.	✓
**Statistical correction:** Describe any statistical corrections (eg, weighting, adjustment for non-representativeness, multiple testing).	✓
